# Meeting the Challenge of Targeting Cancer Stem Cells

**DOI:** 10.3389/fcell.2019.00016

**Published:** 2019-02-18

**Authors:** Alice Turdo, Veronica Veschi, Miriam Gaggianesi, Aurora Chinnici, Paola Bianca, Matilde Todaro, Giorgio Stassi

**Affiliations:** ^1^Department of Surgical, Oncological and Stomatological Sciences, University of Palermo, Palermo, Italy; ^2^Department of PROMISE, University of Palermo, Palermo, Italy

**Keywords:** cancer stem cells, metastasis, anti-cancer therapies, immunotherapy, epigenetic inhibitors

## Abstract

Notwithstanding cancer patients benefit from a plethora of therapeutic alternatives, drug resistance remains a critical hurdle. Indeed, the high mortality rate is associated with metastatic disease, which is mostly incurable due to the refractoriness of metastatic cells to current treatments. Increasing data demonstrate that tumors contain a small subpopulation of cancer stem cells (CSCs) able to establish primary tumor and metastasis. CSCs are endowed with multiple treatment resistance capabilities comprising a highly efficient DNA damage repair machinery, the activation of survival pathways, enhanced cellular plasticity, immune evasion and the adaptation to a hostile microenvironment. Due to the presence of distinct cell populations within a tumor, cancer research has to face the major challenge of targeting the intra-tumoral as well as inter-tumoral heterogeneity. Thus, targeting molecular drivers operating in CSCs, in combination with standard treatments, may improve cancer patients’ outcomes, yielding long-lasting responses. Here, we report a comprehensive overview on the most significant therapeutic advances that have changed the known paradigms of cancer treatment with a particular emphasis on newly developed compounds that selectively affect the CSC population. Specifically, we are focusing on innovative therapeutic approaches including differentiation therapy, anti-angiogenic compounds, immunotherapy and inhibition of epigenetic enzymes and microenvironmental cues.

## Cancer Stem Cells as a Main Determinant of Therapy Refractoriness

Cancer stem cells (CSCs) are defined as being a subpopulation of cells within the heterogeneous tumor mass. This subset of cells is endowed with the ability to self-renew and differentiate into non-CSCs, indicating their capability of reproducing the tumor of origin when transplanted into immunocompromised mice. CSCs are also considered responsible for the metastatic spreading and chemoresistance. Strong evidence suggests that conventional treatments, including radio- and chemotherapy, spare the CSC subset, which is responsible for minimal residual disease (MRD) and cancer relapse (Valent et al., 2012). Indeed, CSCs are characterized by more pronounced levels of drug transporters, enhanced DNA-damage repair mechanisms and the ability to escape the cytotoxic chemotherapy by maintaining a quiescent state. New emerging therapeutic approaches using immunotherapy, anti-angiogenic compounds and/or epigenetic probes aim to overcome the CSC resistance to treatments. CSCs have been thoroughly investigated in the past decades, starting in 1971 when they were observed by Perce and Wallance, who described aggressive undifferentiated cells that are able to generate squamous cell carcinoma *in vivo* (Lobo et al., 2007). CSCs were first identified in Myeloid Leukemia in 1997 and since then they have been proposed to be the tumor initiating cells responsible for disease recurrence and metastasis formation. Bonnet and Dick identified a subpopulation of tumor initiating cells with marked stem-like properties in acute myeloid leukemia (AML). Later, several groups also identified CSCs in solid tumors, including breast, brain, thyroid, melanoma, colon, pancreatic, liver, prostate, lung, head and neck, ovarian, and stomach cancers (Lapidot et al., 1994; Bonnet and Dick, 1997; Al-Hajj et al., 2003; Hemmati et al., 2003; Singh et al., 2004; Collins et al., 2005; Ma et al., 2007; Fukuda et al., 2009; Boiko et al., 2010; Todaro et al., 2010). Based on these studies, a large number of biomarkers can be adopted to identify CSCs (Table 1).

**Table 1:** Expression of CSCs markers according to tumor types.

Tumor type	Cancer stem cell markers
Breast cancer	CD133^+^, CD44^+^, CD24^+^, EpCAM^+^, ALDH^high^
Colon cancer	CD133^+^, CD44^+^, CD24^+^, CD166^+^, EpCAM^+^, ALDH^high^, ESA^+^
Gastric cancer	CD133^+^, CD44^+^, CD24^+^
Glioblastoma	CD133^+^
Head and neck cancer	SSEA-1^+^, CD44^+^, CD133^+^
Leukemia (AML)	CD34^+^, CD38^-^, CD123^+^
Liver cancer	CD133^+^, CD44^+^, CD49f^+^, CD90^+^, ALDH^high^, ABCG2^+^, CD24^+^, ESA^+^
Lung cancer	CD133^+^, CD44^+^, ABCG2^+^, ALDH^high^, CD87^+^, CD90^+^
Melanoma	ABCB5^+^, CD20^+^
Ovarian cancer	CD133^+^, CD44^+^
Pancreatic cancer	CD133^+^, CD44^+^, CD24^+^, ABCG2^+^, ALDH^high^, EpCAM^+^, ESA^+^
Prostate cancer	CD133^+^, CD44^+^, α2β1^+^, ABCG2^+^, ALDH^high^

### Interfering With the Intrinsic Mechanisms of Therapy Resistance in CSCs

Cancer stem cells own a superior capability to survive current therapeutic regimens, meaning that chemo- and radiotherapy are not sufficient to successfully eradicate cancer and are inadequate, especially when the diagnosis occurs at a later stage (Valent et al., 2012; Ajani et al., 2015). Recent evidence showed that the CSC subpopulation is enriched after chemotherapy, suggesting that this subset is responsible for the majority of treatment failure (Visvader and Lindeman, 2008; Alison et al., 2011). Chemoresistance is favored by several mechanisms, among which cellular plasticity. Indeed, Liu et al. (2014) and Luo and Wicha (2019) demonstrated that breast CSCs can switch from proliferating epithelial characteristics to a mesenchymal state which contributes to metastatic dissemination and resistance to therapies.

**FIGURE 1:**
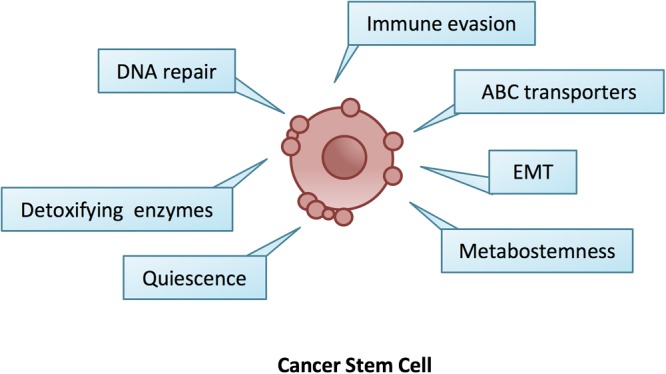
The hallmarks of cancer stem cells. CSCs are endowed with a number of innate ad adaptive responses such as quiescence, EMT, increased DNA repair and detoxifying enzymes, metabostemness, immune evasion and over-expression of ABC transporters, which gave them the ability to survive changes in the microenvironment and anti-cancer therapies.

Nevertheless, the resistance of CSCs to therapy is usually not limited to one drug and this phenomenon referred to as multidrug resistance (MDR) (Efferth et al., 2008). MDR is the result of the endogenous expression of detoxifying enzymes, increased drug efflux pump levels, enhanced DNA repair activity, reduced drug response and activated survival pathways (Singh and Settleman, 2010). These features, combined with the capability of CSCs to evade the immune system, to activate an epithelial to mesenchymal transition (EMT) program and to adapt their metabolism under scarce nutrient conditions, render CSCs almost an imperishable cancer population (Figure 1).

The aldehyde dehydrogenase (ALDH) 1 belongs to the ALDH superfamily, which is composed by 19 enzymes (Hsu et al., 1999). ALDH1 is the main isoform that by oxidizing aldehydes to carboxylic acids and retinol to retinoic acid, allows the detoxification from drugs and reactive oxygen species (ROS) (Singh et al., 2013). ALDH is known to be expressed by normal stem cells, for this reason its activity may be an intrinsic characteristic of CSCs as well. As a result, high levels of ALDH1 activity were found in CSCs, thus representing a reliable marker for the identification of this subset (Charafe-Jauffret et al., 2010). ALDH1 positive cells showed an increased potential of forming xenograft tumors in AML and breast cancer (Cheung et al., 2007; Ginestier et al., 2007). Thereafter, ALDH1^+^ cells from stomach, lung, liver, head and neck, pancreas, cervix, thyroid, prostate, colon, bladder, and ovary tumors were successfully transplanted into mice (Ma et al., 2008a). The implication of the ALDH superfamily in detoxification suggests that these enzymes may have a key role in CSCs’ chemoresistance. Indeed, it has been demonstrated that ALDH expression confers resistance to several chemotherapeutic agents, such as cyclophosphamide, cisplatin, paclitaxel, docetaxel, doxorubicin, and gemcitabine in leukemia, medulloblastoma, adenocarcinoma, colon and breast cancer (Hilton, 1984; Friedman et al., 1992; Tanei et al., 2009; Duong et al., 2012). Moreover, the inhibition of ALDH activity with disulfiram, sorafenib, and sulforaphane can sensitize CSCs to therapy, providing further confirmation of ALDH role in chemoresistance (Rausch et al., 2010).

A large number of studies have demonstrated that the reduction of chemotherapy efficiency is related to an increased drug efflux from cancer cells. This is caused by the aberrant expression of a family of proteins known as ATP-binding cassette (ABC) transporters, which belong to a family of 49 molecules, usually implicated in membrane trafficking using ATP as a source of energy. Among these proteins, ABCB1 (also known as MDR1 or P-gp), ABCG2 (also known as BCRP1), ABCB5 and ABCC1 were largely studied and characterized (Leonard et al., 2003; Lobo et al., 2007). Starting from these premises, it was hypothesized that CSCs may over-express ABC transporters as compared to non-CSCs. Indeed, several groups independently demonstrated that CSCs share features with the Hoechst dye excluding side population (SP), which highly expresses efflux pumps able to induce resistance to harmful toxins and chemotherapeutic compounds (Hirschmann-Jax et al., 2004; Ho et al., 2007). ABC transporters are involved in the resistance to a wide array of drugs. In particular, it was demonstrated that ABCB1 is over-expressed in breast CSCs, causing their resistance to doxorubicin and paclitaxel, and in multiple myeloma stem cells, refractory to carfilzomib (Wright et al., 2008; Hawley et al., 2013). On the other hand, ABCG2 is responsible for the resistance of hepatocellular CSCs to 5-fluorouracile, mephedrone, and cisplatin, whereas ABCB5 was found on circulating melanoma cells resistant to doxorubicin (Frank et al., 2005; Shi et al., 2008). The inhibition of these transporters represents a useful tool to overcome CSCs’ chemoresistance. This was demonstrated by Frank et al., who targeted ABCB5 by way of a specific blocking monoclonal antibody to restore melanoma cells’ sensitivity to doxorubicin, and by Lancet group who demonstrated the sensitizing effect of zosuquidar, a P-gp inhibitor (Frank et al., 2005; Lancet et al., 2009).

The B-cell lymphoma-2 (BCL-2) family plays a pivotal role in regulating cell fate. The pro-survival proteins belonging to this family are BCL-2 itself, B-cell lymphoma extra large (BCL-xL), BCL-2-like-2 (BCL-W), BCL-2-related protein A1A (BCL-A1A), and myeloid cell leukemia sequence-1 (MCL1), whereas the pro-apoptotic molecules include BCL2-associated-X-protein (BAX) and BCL-2 homologous antagonist killer (BAK) (Kelly and Strasser, 2011). Among these molecules, BCL-2 was found over-expressed in breast CSCs, while both BCL-2 and BCL-xL were found up-regulated in leukemia CSCs (Konopleva et al., 2002; Madjd et al., 2009). The role of the BCL-2 family has been further elucidated by Strasser et al. (1990) who demonstrated that BCL-2 over-expression promotes tumorigenesis. Consequently, the inhibition of BCL-2 downstream pathways caused an increased sensitization to chemotherapy in colon and hepatocellular CSCs (Todaro et al., 2007; Ma et al., 2008b).

*In vitro* evidence suggests that CSCs are slow-cycling if compared to non-CSCs (Viale et al., 2009). Interestingly, quiescence makes CSCs less sensitive to cell-cycle directed therapies such as vinca alkaloids, which prevents the polarization of microtubules and taxanes, known to stabilize existing microtubules (Gascoigne and Taylor, 2009).

Chemotherapeutic agents and radiotherapy are used in clinical setting to induce DNA damage. Of note, CSCs do not respond to therapy due to increased activity of DNA repair machinery (Bao et al., 2006; Eyler et al., 2008; McCord et al., 2009; Ropolo et al., 2009). In fact, in glioma and breast CSCs, a higher phosphorylation of DNA repair proteins was observed, in particular in ATM, CHK1, and CHK2 (Eyler and Rich, 2008; Gallmeier et al., 2011; Maugeri-Sacca et al., 2011). Moreover, ovarian and lung CSCs are enriched after cisplatin treatment, a further indication that chemotherapy is limited to kill the proliferating fraction of the tumor bulk (Levina et al., 2008; Rizzo et al., 2011).

Furthermore, it has been demonstrated that chemotherapy induced damage stimulates glioblastoma multiforme and bladder CSCs to divide and thus to repopulate tumor bulk (Chen et al., 2012; Kurtova et al., 2015). On the other hand, this induced proliferation may be exploited to increase the efficacy of therapeutic regimens (Saito et al., 2010). Interestingly, the induction of CSC differentiation by using the bone morphogenic protein 4 (BMP4) renders these cells more susceptible to standard and targeted anti-cancer therapies (Lombardo et al., 2011). Furthermore, the all-*trans* retinoic acid is among the most common drugs used to cause differentiation of stem cells particularly in acute promyelocytic leukemia (Nowak et al., 2009). Inhibitors of epigenetic modulators such as DNA methyltransferase 1 (DNMT1), histone deacetylases (HDACs) and bromodomain and extra-terminal (BET) inhibitors have shown capabilities to function as differentiation therapies for CSCs in various tumor types (Toh et al., 2017).

Additionally, one cancer hallmark is the activation of angiogenesis, which concurs with the nurture of the tumor mass by stimulating *de novo* vessels formation (Hanahan and Weinberg, 2011).

### Targeting the ‘Metabostemness’

Compelling evidence suggests that stem-like features can be acquired as a result of metabolic shifts, which are able to render normal stem cells or differentiated cancer cells more susceptible to epigenetic reprogramming. These cells are thus more likely to move up the cancer cell hierarchy by their expression of pluripotent genes. The metabolic insults, able to induce this reprogramming into CSCs in the context of a pre-malignant tumor, are collectively termed ‘metabostemness’ (Menendez and Alarcon, 2014). Consistently, some of the intermediates deriving from mutated metabolic enzymes, involved in glycolysis, tricarboxylic acid cycle, oxidative phosphorylation (OXPHOS) and mitochondrial fatty acid oxidation, act as oncometabolites for DNA and histones epigenetic modifications by driving tumorigenesis (Menendez and Alarcon, 2014). For this reason, targeting metabolic processes may represent a successful strategy. In particular, in most cases OXPHOS is the preferential source of energy rather than glycolysis, probably because of the low levels of glucose in tumors. Moreover, increased OXPHOS is a hallmark of resistance to chemotherapy (Lee et al., 2017). Therefore, it is not surprising that the targeting of OXPHOS *via* the BCL-2 inhibitor venetoclax in combination with the hypomethylating agent azacitidine was able to impair leukemia stem cells (LSCs) proliferation and metabolic activity (Jones et al., 2018; Pollyea et al., 2018). Accordingly, the OXPHOS inhibitor salinomycin was able to kill breast CSCs (Gupta et al., 2009). Interestingly, it has been shown that, during relapse, LSCs are able to rescue OXPHOS levels after amino acid depletion thanks to increased mitochondrial fatty acid oxidation (FAO) (Jones et al., 2018). FAO can also be promoted by the crosstalk with adipose tissue, which fuels LSCs metabolism by acting as a niche and promoting LSCs chemoresistance (Ye et al., 2016). In addition, the targeting of lipolysis, and in particular of COPI-Arf1 complex, was shown to be a promising tool for the eradication of CSCs in adult Drosophila (Singh et al., 2016). The crucial role of mitochondria in CSCs impelled several groups to develop therapeutic strategies aimed at their targeting (Skoda et al., 2018). Notably, mitochondrial biogenesis can be abrogated through the estrogen-related receptor α inhibitor XCT790 (Deblois and Giguere, 2011; Deblois et al., 2013), whereas their fission can be impaired thanks to the dynamin-related protein 1 (DRP1) inhibitors Mdivi-1 and P110 (Xie et al., 2015). In addition, it has been demonstrated that DRP1 activation may be promoted by the interaction between cyclooxygenase-2 (COX-2) and mitochondria. For this reason COX-2 inhibitors, resveratrol and celecoxib, were repositioned as mitochondrial fission inhibitors (Guo et al., 2015; Cilibrasi et al., 2017). Inhibitors of mitochondrial respiration were used to target pancreatic CSC subset (Sancho et al., 2016). Likewise, the inhibition of the mitochondrial complex I through the repositioning of the antidiabetic drug Metformin was recently proposed with encouraging results (Wheaton et al., 2014).

## Cancer Stem Cells, Tumor Microenvironment, Angiogenesis and Metastasis: How to Disrupt This Intricate Network?

**FIGURE 2:**
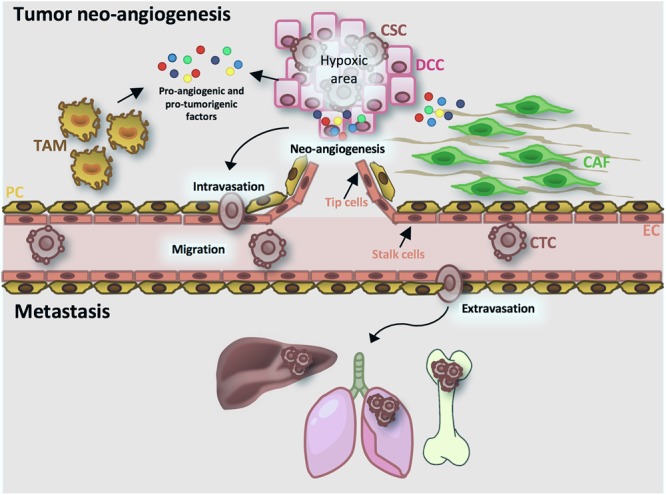
Tumor angiogenesis and metastatic process. Cancer cells secrete pro-angiogenic and pro-tumorigenic factors (MMPs, VEGF-A, HIF-1A, cytokines, chemokines, growth factors). VEGF-A activates endothelial cells (ECs) in tip cells, that direct the sprouting vessels, and stalk cells, implicated in vessel stability. Moreover, cancer cells-released cytokines activate cancer-associated fibroblasts (CAFs) and activated tumor-associated macrophages (TAMs), that in turn favor the intravasation of cancer stem cells (CSCs). Circulating cancer cells (CTCs) through the bloodstream reach target organ, extravasate and start to proliferate and disseminate. CSC, cancer stem cell; CTC, tumor circulating cells; DCC, differentiated cancer cell; CAF, cancer associated fibroblast; TAM, tumor associated macrophage; EC, endothelial cell; PC, pericyte cell.

Angiogenesis is a multistep physiological process, characterized by the formation of new vessels from preexisting ones, which governs many biological activities, such as development and tissue repair. In order to maintain tissue homeostasis, angiogenesis is tightly regulated by a balance between pro- and anti-angiogenic factors (Hanahan and Folkman, 1996). In pathological conditions, such as cancer, this balance is destroyed favoring the secretion of pro-angiogenic factors. The term “tumor angiogenesis” was used for the first time by Folkman (1971) to point out the sprouting of cancer-associated neo-vessels from existing vessels that are in close proximity. Proliferating cancer cells require oxygen and high amount of nutrients, leading to the formation of hypoxic areas in the innermost part of the tumor. Under hypoxic condition, CSCs increase hypoxia-inducible factor-1 (HIF-1) expression and activate the HIF-1 pathway, enhancing the secretion of many angiogenic growth factors (Pugh and Ratcliffe, 2003; Gilbertson and Rich, 2007). In particular, high levels of vascular endothelial growth factor-A (VEGF-A) recruit VEGF receptors (VEGFRs)-expressing endothelial cells (ECs), named tip cells. After VEGF-A binding, tip cells up-regulate cell proliferation, cytoskeleton remodeling and migration pathways (MAPK, PI3K/AKT, RhoA), sprout toward tumor cells and activate the adjacent ECs (stalk cells) to form new tumor vessels (Ricciuti et al., 2017). In addition to ECs, CSCs’ secreted cytokines prime the microenvironment (tumor microenvironment, TME) and recruit myeloid cells to fuel cancer progression. In particular, cancer-associated fibroblasts (CAFs) and activated tumor-associated macrophages (TAMs) secrete high levels of metalloproteases (MMPs), growth factors and interleukins to sustain angiogenesis and to promote CSC invasion (Bhowmick et al., 2004; Crawford and Ferrara, 2009; Owen and Mohamadzadeh, 2013). Furthermore, it has been reported that *de novo* vessel formation may be boosted by CSCs from different tumor types; this process is termed vascular mimicry (Weis and Cheresh, 2011). It has been described that breast and glioblastoma CSCs could give rise to both ECs and pericytes supporting tumor growth and progression (Bussolati et al., 2009; Ricci-Vitiani et al., 2010; Wang et al., 2010; Cheng et al., 2013). Unlike normal vasculature, tumor vessels are tortuous and more permeable due to the lower presence of pericytes (Jain, 2005; Sawada et al., 2012). This “leakiness” reduces the capacity of chemotherapeutic agents to target cancer cells and facilitate the intravasation of metastatic cancer cells. These circulating tumor cells (CTCs) possess a CSC-like phenotype, characterized by a high expression of EMT-related genes (Burgess, 2013; Grillet et al., 2017). Although many cancer cells are able to intravasate, only few cells survive in the bloodstream and extravasate, activating a mesenchymal-epithelial transition program (Tam and Weinberg, 2013). The persistence of extravasated cells requires the presence of a favorable host microenvironment (metastatic niche) and the escape from immune cell surveillance. For these reasons, tumor cells remain in a dormant state, which can last many years, and, after the release of molecules and growth factors by the metastatic niche, they restart to proliferate and disseminate (Gao et al., 2012; Giancotti, 2013) (Figure 2).

### Targeting Tumor Angiogenesis and Metastasis

The possibility of specifically blocking tumor angiogenesis and the metastatic process could have a clinical impact on cancer patients’ outcome. Bevacizumab is a humanized monoclonal antibody that binds to VEGF, impairing VEGF/VEGFR interaction, approved in 2004 by the Food and Drug Administration (FDA) for the treatment of metastatic colorectal cancer (CRC) in combination with standard therapy (Ferrara et al., 2004; Hurwitz et al., 2004). Then, it was approved for the treatment of other metastatic cancers, among which non-squamous NSCLC and cervical cancer (Tewari et al., 2014; Lin et al., 2016). In 2008, bevacizumab was approved for the treatment of metastatic Her2 negative breast cancer in combination with paclitaxel. However, other studies did not show a significant overall survival (OS) and the FDA withdrew the approval in 2011 for breast cancer treatment (Miller et al., 2007; Aalders et al., 2017). Conversely, the European Medicines Agency maintains bevacizumab approval in combination with chemotherapy. Another strategy to inhibit tumor angiogenesis is the use of tyrosine kinase inhibitors, such as sorafenib and sunitinib. Sorafenib is an inhibitor of VEGFR-1,-2,-3 and PDGFR-β, approved for the treatment of metastatic renal cell carcinoma and unresectable hepatocellular carcinoma (Wilhelm et al., 2006; Escudier et al., 2007), whereas sunitinib blocks VEGFR-2 and PDGFR phosphorylation and is used for gastrointestinal tumor and metastatic renal cell carcinoma (Sun et al., 2003; Motzer et al., 2007). Although anti-angiogenic therapy may potentially have clinical implication, the increase of OS is insufficient. This is probably due to (i) acquired resistance (Lu et al., 2012); (ii) the increment of tumor hypoxia (Erler et al., 2009) and (iii) the diminished delivery of chemotherapeutic agents (Jain, 2005).

Metalloproteases are crucial mediators of tumor angiogenesis and cell migration (Kessenbrock et al., 2010). Although many MMP inhibitors have been developed and many clinical trials have been conducted, none of these have increased patients’ OS (Coussens et al., 2002; Winer et al., 2018). On the contrary, MMP inhibitors have numerous side effects, due to the MMP’s role in numerous physiological processes. In order to obtain clinical benefits, inhibitors should be highly selective for MMPs that drive tumor progression.

The dysregulation of stem cell-specific signaling pathways, such as Notch, Wnt and Hedgehog, could reduce metastatic progression. In glioma patients, the use of a gamma secretase inhibitor (RO4929097) reduced CSC number; unfortunately the prolonged use of this inhibitor led to the acquisition of angiogenesis-mediated resistance (Pan et al., 2016; Xu et al., 2016). Vismodegib, an inhibitor of a component of the Hedgehog pathway, Smoothened, was used in combination with gemcitabine in pancreatic cancer, without affecting CSC number (Catenacci et al., 2015). In order to increase the efficacy of these inhibitors that target CSCs and block metastasis development, further studies must be carried out especially to reduce the side effects.

Cytokines, chemokines and growth factors secreted by TME cells enhance the migration capacity of cancer cells and promote angiogenesis (Wakefield and Hill, 2013; Scala, 2015; Gaggianesi et al., 2017). Therefore the inhibition of their receptors could have clinical benefits. In fact, reparixin, an inhibitor of IL-8 receptor CXCR1, reduced the breast CSC population and lung metastases (Ginestier et al., 2010) and is used in combination with paclitaxel in an ongoing clinical trial in triple negative breast cancer patients (Marcucci et al., 2016).

## Harnessing the Immune System to Flush Out and Eradicate Cancer Stem Cells

The new frontier of cancer treatment is aimed at strengthening the immune system’s defenses against cancer cells. In the last decade the remarkable progress made on immunotherapy heralded an impressive novelty in the management of patients affected by a variety of cancers. The results obtained by immune-based therapies in terms of durable objective response rate exceeded expectations and it is no wonder that the scientists James Allison and Tasuku Honjo were recently awarded the 2018 Nobel prize in medicine for their pioneering discoveries in immunotherapy. Their studies were different, although based on the same principle: to fight cancer by harnessing the immune system.

Several compounds based on the inhibition of immune checkpoints have been approved by the FDA since 2011. Ever since, the most promising of these therapies have been antibodies targeting the cytotoxic T lymphocyte-associated protein 4 (CTLA-4) or the programmed cell death 1 (PD-1) pathway, administered as single therapy or in combination.

The role of CTLA-4 as a negative regulator of T cell activation was discovered in the laboratory of James Allison and Jeffrey Bluestone. To induce antitumor responses, T cells are initially activated in the lymph node in two subsequent steps (i) engagement of T cell receptor (TCR) with a tumor antigen MHC complex on antigen presenting cells (APCs) and (ii) binding of CD28 to the costimulatory molecule B7. Following T cell activation, CTLA-4 translocates from the intracellular compartment to the cells’ surface to compete with the costimulatory molecules, causing the inhibition of T cell proliferation. The blockade of this essential immune checkpoint with monoclonal antibodies enables T cells to active, expand and reach the tumor burden, where they can find the cognate antigen presented by cancer cells (Ribas and Wolchok, 2018).

Otherwise, Tasuku Honjo demonstrated that TCR engagement at the tumor site causes the expression of the PD-1 receptor that binds the PD-1 ligand (PD-L1) on cancer cells, causing the exhaustion of T cells and hampering the antitumor cytotoxic T cell responses (Okazaki et al., 2013).

These two mechanisms are generally implemented to impede the overstimulation of the immune system but in the context of cancer, they become detrimental for cancer cell elimination. Nevertheless, an immune checkpoint blockade could be exploited to potentiate the antitumor immune response.

Ipilimumab was the first CTLA-4 inhibitor that entered the clinic and was approved by the FDA in 2011. A substantial portion of advanced melanoma patients treated with ipilimumab had a durable response that was unluckily accompanied by toxicity, such as colitis and the inflammation of endocrine glands. Nivolumab and pembrolizumab were the first anti-PD-1 compounds approved by the FDA for melanoma (2014) and NSCLC (2015) followed by the approval of anti-PD-L1 antibodies, atezolizumab, avelumab, and durvalumab. Interestingly, the anti-PD-1 pathway inhibitors were approved for the first time based on their genetic background as for example, the presence of unstable microsatellite rather than the cancer type. Objective response rate was high varying from 15% for head and neck, gastroesophageal, bladder and urinary tract cancers and reaching almost 90% for Hodgkin’s disease. Of interest, an ongoing phase 2 clinical trial is assessing the optimal adaptive dosage of an ipilimumab and nivolumab combination in metastatic melanoma patients (NCT03122522). Moreover, other promising results have been achieved by preclinical studies that show a synergistic effect of anti-HER2 antibodies and immune checkpoint inhibitors in breast cancer (Su et al., 2018).

Albeit immune checkpoint inhibitors are considered the spearhead of immunotherapy against cancers that show an high mutation burden, the expected accumulation of neoantigen and the high PD-1/PD-L1 expression (Bailey et al., 2018) may not produce a greater antitumor response (Gide et al., 2018).

**FIGURE 3:**
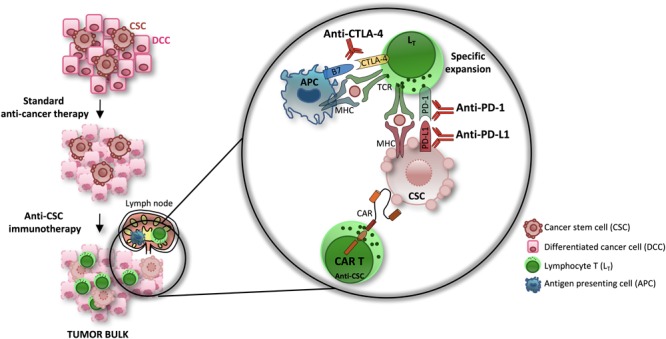
The efficacy of immune-based therapies in the eradication of CSCs. The standard anti-cancer therapies are able to affect differentiated cancer cells (DCCs) while sparing CSCs. Novel immunotherapy approaches have shown promising therapeutic efficacy in several type of cancers. Combinations of checkpoint inhibitors (CTLA-4 and PD-1/PD-L1 pathway inhibitors) and CAR T cell transfer, in particular, efficiently eliminate the CSCs subpopulation.

According to the new iRECIST criteria of tumor response following the administration of immunotherapy (Seymour et al., 2017), patients undergoing treatment resistance may experience pseudoprogression or hyperprogression, which consists in the initial increase of tumor volume followed by its decrease or in a faster progression of the disease as compared to the predicted rate, respectively (Champiat et al., 2017; Siefker-Radtke and Curti, 2018). The mechanisms of resistance to immune checkpoint inhibitors may be caused by the persistence of a subpopulation of CSCs. Indeed, the activation of transcriptomic profiles characterized by genes involved in EMT, angiogenesis and stemness causes the lack of T cell recognition and immunotherapy refractoriness (Spranger et al., 2015; Hugo et al., 2016). Indeed, CSCs can evade the immune system (Lee et al., 2016; Hsu et al., 2018) mainly due to the high expression levels of PD-L1 (Wu et al., 2017), the down-regulation of molecules involved in the presentation of the antigen to T cells (Bruttel and Wischhusen, 2014) and their capacity to promote the formation of an immune suppressive microenvironment (Jachetti et al., 2015; Sorrentino et al., 2018; Szarynska et al., 2018). On the other hand, the high levels of PD-L1 expressed by the CSCs render them potentially susceptible to treatments with checkpoint inhibitors, which can be combined with other immune-based therapies for an effective response (Figure 3).

For instance, in a syngeneic melanoma mouse model, the combination of immune checkpoint inhibitors (PD-L1 inhibitors and CTLA-4 inhibitors) with CSC lysate-pulsed dendritic cells (DCs) vaccine augmented T cell antitumor response and led to tumor regression (Zheng et al., 2018).

The chimeric antigen receptor (CAR) T cell transfer is currently being investigated (Gargett et al., 2016) and holds great promise in the treatment of liquid and solid malignancies (Grupp et al., 2013; Mount et al., 2018). CAR T cells are constituted by an antigen receptor linked by a single chain fragment to an intracellular domain, usually supplemented with a co-stimulatory molecule. CAR T cells offer an exceptional substrate for the development of selective CSCs therapies, being potentially able to recognize any antigen exposed on the surface of CSCs (Guo et al., 2018). A case report has recently been described by Feng et al. (2017), which shows the efficacy of the subsequent infusion of CAR T anti-EGFR and anti-CD133, a well known marker that identifies CSCs, in a patient affected by cholangiocarcinoma. The CAR T-EGFR and CAR T-CD133 are currently under clinical evaluation (NCT01869166 and NCT02541370). Moreover, CAR T cells, which target the CSC marker EpCAM, reduced prostate cancer progression in preclinical models (Deng et al., 2015). Thus, the CAR T cell-based therapies offer the opportunity to specifically eliminate the CSC subpopulation and are a valid alternative to checkpoint inhibitors, in a subset of cancer with paucity of neoantigens expression. Additionally, CAR T cells transfer could strengthen the efficacy of CTLA-4/PD-1 pathway inhibitors and targeted therapies.

Hence, contrary to the targeted therapies, which are almost mutation-related and could induce the reactivation of alternative survival pathways, immunotherapy offers the opportunity to achieve long-lasting responses in a broad range of tumor types, by overcoming the highly adaptive behavior of CSCs.

## Epigenetic Reprogramming and Cancer Stem Cells

Dynamic epigenetic reprogramming of the CSC subpopulation adds a further layer of inter- and intra-tumor heterogeneity to the complexity of tumors, which represents a hurdle for successful therapies. Epigenetics is the study of heritable changes and phenotypes not encoded in DNA (Dawson, 2017). The epigenetic enzymes responsible for histone modifications (writers, erasers, and readers) and DNA methylation (DNMT) have been extensively described (Arrowsmith et al., 2012). The histone methylation and acetylation are catalyzed by histone methyltransferases (HMTs) and histone acetyltransferases (HATs), while the histone demethylation and deacetylation are catalyzed by the histone demethylases (HDMs) and HDACs, respectively. Acetylated histones tend to be less compact and more accessible to RNA polymerase and transcriptional machinery, thereby enabling the transcription of nearby genes. Methylated histones can be either repressive or activating, depending on the site and degree of methylation. In particular, histone H3/H4 acetylation (H3Ac, H4Ac) and H3 lysine 4 methylation (H3K4me) are generally associated with active transcription, while histone H3 lysine 9 and 27 methylation (H3K9me, H3K27me) are commonly linked to gene repression (Bird, 1986; Jones and Takai, 2001; Venters and Pugh, 2007; McCabe et al., 2009). The well-known “histone code” hypothesis is based on the knowledge that different patterns of histone modifications on each histone determine the ultimate transcriptional event, either gene expression or silencing (Strahl and Allis, 2000). Several interrelated molecular mechanisms contribute to epigenetic gene regulation, such as chromatin remodeling via ATP-dependent processes and exchange of histone variants, regulation by non-coding RNAs, methylation and related modifications of cytosines on DNA, as well as covalent modification of histones. Local chromatin state at gene promoter is governed by DNMT and posttranslational histone modifications, thus playing an essential role in transcription regulation. DNMT1, DNMT3A, and DNMT3B are responsible for the methylation of the CpG islands, CpG-dense regions that are included in the majority of human gene promoters. While the unmethylated status of CpG islands is aimed to maintain promoter chromatin in a transcriptionally permissive state, their methylation is linked to gene silencing (e.g., X-chromosome inactivation, tumor suppressor gene silencing in cancers). The chromatin remodeling complexes, including the SWI/SNF complex, are at least five families that use ATP-hydrolysis to modify chromatin structure and remodel nucleosomes. Polycomb repressive complexes (PRC1 and PRC2) are epigenetic repressors of transcriptional programs fundamental for the cell’s identity, development, differentiation and lineage specification, by catalyzing the trimethylation of histone 3 lysine 27 (H3K27me3) (Di Croce and Helin, 2013). Recently, it has been demonstrated that EZH2, the functional enzymatic component of the PRC2, is required for stable self-renewal and differentiation not only in mouse but also in human embryonic stem cells (Collinson et al., 2016).

Epigenetic alterations, including DNMT and histone modifications, are a key manifestation of the stem cells’ differentiation into various tissue subtypes. The increasing number of recently discovered mutations in epigenetic regulators has shed new light on the importance of epigenetic dysregulation in tumor initiation and in the biology of CSCs. These may originate from a deregulated epigenetic reprogramming, which leads to the loss of differentiation genes and to the reestablishment of stem cell-specific characteristics. Epigenetic mechanisms play an important role in endowing stem cell characteristics to cancer cells. This is well established in many types of cancer, as: (1) CSC markers are directly regulated by epigenetic modifications (i.e., CD133 and DCLK1) (Yi et al., 2008; Vedeld et al., 2014); (2) CSCs exhibit mutations in chromatin remodeler components (loss of function mutations of PRC2 complex and deregulation of EZH2) (van Vlerken et al., 2013); (3) EMT, which confers cells with tumor-initiating capabilities and CSC properties (Mani et al., 2008), is finely controlled by epigenetic mechanisms (Kanwar et al., 2010; Beck et al., 2015; Avgustinova and Benitah, 2016).

**FIGURE 4:**
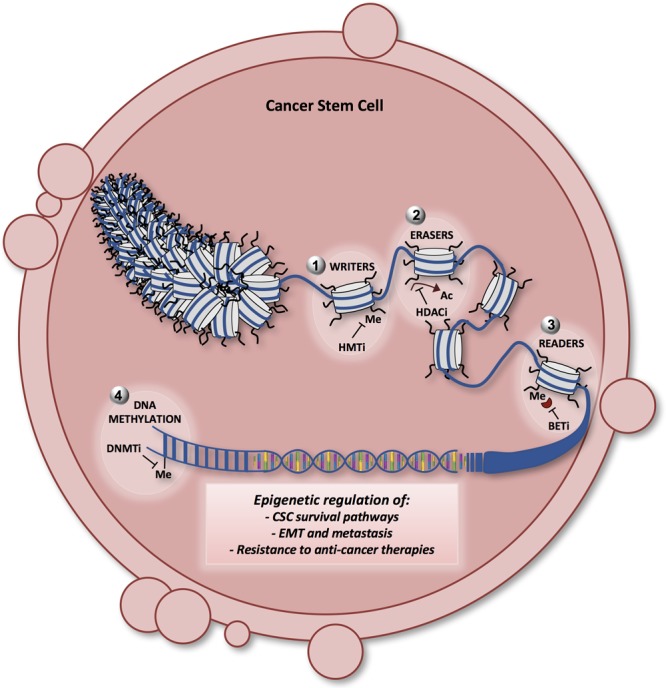
Model showing the different layers of epigenetic regulation in CSCs and the potential therapeutic approaches. The chromatin fiber and the nucleosome are represented in the nucleus of a cancer stem cell. Epigenetic enzymes [writers (1), erasers (2), readers (3), and DNA methyltransferases (4)] are the principal actors in regulating the key survival pathways in CSCs, such as the Notch, Wnt and Hedgehog signaling. Moreover, epigenetic alterations guide the epithelial-mesenchymal transition (EMT) and the aberrant process of metastatization in CSCs, contributing to CSC resistance to therapy. Many of the latest generation compounds (epigenetic probes) have been designed to target the epigenetic enzymes involved in the CSC survival, maintenance, EMT and metastasis. DNMTi (DNA methyltransferase inhibitors): decitabine, azacitidine; HMTi (histone methyltransferase inhibitors) such as EZH2, DOT1L, and SETD8 inhibitors; HDACi (histone deacetylase inhibitors): vorinostat, romidepsin; BETi (bromodomain inhibitors): JQ1, I-BET762. Me, methylation; Ac, acetylation.

This link between epigenetics and CSCs suggests that epigenetic alterations may be key therapeutic targets in this abnormal subpopulation. Furthermore, the development of specific epigenetic enzymes inhibitors has been a promising area of drug discovery, due in part to the “druggability” of these critical regulators. Therefore, an extensive investigation of the epigenetic enzymatic activities that are critical for the reprogramming of CSCs toward differentiation may be crucial for the tailoring and designing of new therapeutic strategies against a variety of deadly tumors. Hence, epigenetics enzymes are fundamental in regulating survival pathways, EMT, metastatic phenotype and chemoresistance in CSCs (Figure 4).

### CSC Formation and Maintenance

Of note, many epigenetic mechanisms that promote the acquisition of uncontrolled self-renewal and CSC formation are based on driver mutations that have been found in principal epigenetic regulators, in both chromatin-related driver genes and DNA-methylation-related genes (Wainwright and Scaffidi, 2017).

LSCs bear the fusion protein product of the *KMT2/MLL* gene. This gene encodes for a HMT involved in many biological processes. Importantly, the MLL fusion proteins have been associated with an oncogenic role due to their ability to initiate the tumorigenic process in both AML and acute lymphoblastic leukemia (ALL) cells (Cozzio et al., 2003; Krivtsov et al., 2006; Somervaille et al., 2009).

In about 33% of pediatric glioblastoma patients, gain-of-function mutations have been identified in the gene encoding for histone H3. The most represented alteration is a K27M substitution, which leads to the impaired functions of the PRC2 complex and a lack of gene repression, which in turn leads to the aberrant activation of oncogenic programs and self-renewal ability (Lewis et al., 2013). DNMTs are mutated in 25% of AML patients. These mutations hamper the enzymatic activity and leads to the propagation of pre-LSCs.

Importantly, the dynamic cooperation between the genetic and epigenetic alterations in cancer initiation and promotion has been supported by recent evidence, especially in CRC model.

DNA methyltransferases have been shown to play a key role in the initiation and progression of CRC. Many tumor suppressor gene promoters are hypermethylated in CRC (*MLH1, RB, P16, RARB, SFRP*). The expression and the activity of the DNMTs seem to be controlled by *APC* mutation, a driver event in CRC (Hammoud et al., 2013), confirming once again that genetic and epigenetic interactions may cooperate to induce tumor initiation and progression. Specifically, it has been shown that the expression levels of DNMT1 are higher in CRCs compared to normal controls, suggesting that the elevated levels of this DNA methyltransferase may determine a dysregulation in the methylome by suppressing the transcription of the tumor suppressor genes. Moreover, this supports the hypothesis that deregulation of DNMT in CR-CSCs could be a crucial event during cancer progression.

Crucial pathways involved in CSC maintenance, such as Wnt/β-catenin, Notch and Hedgehog signaling pathways are finely regulated by epigenetic mechanisms. These pathways in physiological conditions control self-renewal and development in embryonic and adult stem cells. DNMT, aberrant histone modification and also non-coding RNA have been identified as epigenetic aberrations in the main regulators of these pathways in CSCs. For instance, aberrant DNMT silences Wnt inhibitory factor genes with a tumor suppressor role, such as *WIF-1, AXIN-2, SFRP-1*, and *DKK1* (Suzuki et al., 2004). The promoter of *DKK1* is also silenced by decreased acetylation of H3K16 and increased H3K27 trimethylation (Hussain et al., 2009). In multiple myeloma cells, an enhanced histone acetylation has been found at the promoter region of *JAGGED2*, a Notch receptor ligand, leading to the activation of Notch signaling by overexpression of its ligand (Ghoshal et al., 2009). The histone methylation of H3K27 is inhibited on the promoters of two Notch signaling target genes, *HES1* and *HES5*. This is accomplished by the serine-threonine kinase receptor-associated protein (STRAP), which interacts with PRC2 complex components, thus leading to gene activation in CRC. SNF5, a member of a chromatin remodeler complex SWI/SNF, binds directly Gli1, which is the down-stream effector of the Hedgehog signaling pathway, leading to a repression of the target genes transcription (42). Indeed, in human malignant rhabdoid tumors inactivation of SNF5 results in an aberrant activation of Hedgehog signaling. Moreover, HDAC1 is required to transcriptionally activate Gli1 and Gli2. However, this inhibitory mechanism is hampered by the frequent somatic mutations in *REN* gene, which encodes for the E3-ubiquitin ligase complex that mediates the degradation of HDAC1 (Di Marcotullio et al., 2004; Canettieri et al., 2010). Aberrant DNA hypomethylation of the Sonic Hedgehog ligand promoter is responsible for the pathway activation.

Therefore, the integration of genetic and epigenetic mechanisms disrupts the balance between self-renewal and pro-differentiation stimuli thus generating an aberrant program that sustains CSC survival.

### EMT, Metastasis, and Resistance to Therapies in CSCs

The concept of CSCs in the maintenance and progression of many types of cancer is now well accepted and continues to evolve (Todaro et al., 2007, 2014; Kemper et al., 2010). This cell status is dynamic during cancer progression as it is mainly affected by genetic and epigenetic changes and influenced by the TME. Another characteristic of CSCs is their ability to invade and metastasize by acquiring the EMT phenotype that can be determined by examining the expression of E-cadherin (CDH1) and vimentin, which represent the effectors for Wnt and Notch signaling. It has been reported that Wnt/β-catenin signaling plays a critical role in regulating growth and maintenance of CR-CSCs (Kanwar et al., 2010). In particular, our group identified CD44v6 as a marker of metastatic potential that defines the CR-CSC subpopulation (Todaro et al., 2014). In CR-CSCs the up-regulation of Wnt signaling is correlated with a higher CD44v6 expression, suggesting that this population may retain metastatic traits and chemoresistance.

Many different epigenetic mechanisms have been linked to the activation of an uncontrolled EMT process. The loss of E-cadherin can be defined as a hallmark of EMT given the lack of the cell–cell adhesion. Of note, DNMT of the *CDH1* promoter by recruiting HDACs to the promoter site results in histone deacetylation and transcriptional silencing. Furthermore, EZH2 and the PRC2 complex mediate the histone methylation of the *CDH1* promoter, repressing its expression (Cao et al., 2008).

MiR-200 family members have been associated with a role in repressing EMT and invasion through a direct binding to ZEB1 and ZEB2 (zing finger E-box-binding homeobox 1 and 2), which are two transcription factors. Epigenetic silencing of these miRNAs by DNMT and H3K27 tri-methylation induces the acquisition of both an EMT-like and CSC phenotype (Tellez et al., 2011).

One of the most common mechanisms of drug resistance, subjected to epigenetic regulation in CSCs, is mediated by a pronounced expression of the drug efflux transporters, such as the ATP-binding cassette family (ABCG2, MDR1, MRP1). Decreased HDAC1 levels and increased histone acetylation and phosphorylation are responsible of an enhanced expression of ABCG2 (To et al., 2008).

### Alternative Epigenetic Mechanisms of CSCs Regulation

On one hand, it is well known that the addition or removal of epigenetic marks on the histones of the nucleosomes play a crucial role in regulating the gene expression of oncogenic drivers or oncosuppressors (Louis and Shohet, 2015). On the other hand, oncogenes and tumor suppressors can themselves be activators of epigenetic mechanisms fundamental in CSCs by the induction of a “non-canonical” epigenetic program. Indeed, recent data have demonstrated that MYC favors a stem cell-like phenotype in mammary epithelial cells and induces an alternative epigenetic program, supported by the activation of *de novo* enhancers and repression of lineage-specifying transcription factors, which causes loss of cell identity and the activation of oncogenic pathways (Poli et al., 2018). Moreover, HMTs can methylate non-histone proteins such as the pivotal tumor suppressor gene *TP53*. It has been demonstrated that the tumor suppressor function of WT p53 is inhibited by repressive epigenetic pathways. p53 and “stemness” may be considered as conceptual antagonists. p53 suppresses self-renewal and promotes differentiation of adult stem cells. Inactivation of p53, by deletion, mutation, or expression of dominant-negative isoforms of p53 family members, enriches stem cell populations including CSCs (Molchadsky and Rotter, 2017). Some HMTs (SETD8 and SMYD2) have been found to regulate the methylation of non-histone proteins in particular p53 in lysine residues. These modifications such as the monomethylation on lysine 370 and lysine 382 of p53 (p53K370me1 and p53K382me1) have been associated with a pro-tumorigenic function (Zhu et al., 2016; Veschi et al., 2017). Further studies are needed to better elucidate these mechanisms and their targeting as a therapeutic approach in CSCs.

### Treatments That Target Epigenetic Modifications in CSCs

The dynamic nature of epigenetics indicates that it may be possible to alter cancer-associated epigenetic states through direct manipulation of the molecular factors involved in this process. Currently, the major challenge in epigenetic drug discovery is to identify selective compounds with significant *in vitro* cellular activity at nM concentrations and well tolerated *in vivo*. Recently, mostly by using high throughput screening approaches, many studies identified and characterized new epigenetic regulators and their roles in various cancers. These findings represent the translational basis for the initiation of clinical trials in the area of specific epigenetic target classes. HDACs and DNMTs were the first epigenetic targets to be approved for cancer application by the FDA, but more recently additional families of epigenetic regulators have been the subject of intense studies, such as, methyltransferases (EZH2, SETD8, DOT1L, PRMT5), demethylase (LSD1, KDM4B), and BET proteins. Some potent inhibitors are now being studied in a clinical setting, more specifically in hematological and solid tumors. Early results are encouraging, despite relevant toxicity.

Histone deacetylases are key regulators of histone acetylation levels and are mostly associated with enhanced gene transcription. HDACs remove acetyl groups on histones’ lysine residues and maintain cell balance by opposing the function of the HATs. Despite promising anti-cancer data from clinical trials, HDAC inhibitors need to be considered as pan-inhibitors with associated side effects, although increasing efforts have been made to develop selective HDAC inhibitors. Vorinostat (SAHA) represents the first FDA approved pan-HDAC inhibitor that targets HDAC1-3 and 6. Currently, there are 6 clinical trials using Vorinostat targeting refractory or recurrent pediatric cancers and adult tumors. Romidepsin is an FDA approved selective HDAC1/2 inhibitor that is well tolerated in clinical trials for advanced pediatric and adult tumors (Children’s Oncology et al., 2006; Amiri-Kordestani et al., 2013). DNA demethylating or hypomethylating agents, such as DNMTs inhibitors (DNMTi, azacitidine and decitabine), are currently in clinical phases I and II for a variety of tumors, including CRC.

The chromatin readers (BET family) recruit additional chromatin modifiers and remodeling enzymes, which serve as the effectors of the modification. For instance, acetylated histones serve as docking sites for bromodomain containing proteins (Dhalluin et al., 1999; Dey et al., 2003). Thus, the histone code imparts a tertiary level of genomic control beyond the DNA sequence and corresponding transcription factors (He et al., 2013). BET inhibitors have been demonstrated to successfully target CSCs in MLL-driven ALL and in other cancers. Among the first selective and more efficacious BET inhibitors, JQ1 is able to target c-MYC in many different cancers and I-BET762 is in Phase I-II clinical trial for NUT midline carcinoma, neuroblastoma and other tumors^1^ (Filippakopoulos et al., 2010; Filippakopoulos and Knapp, 2014). However, nowadays many deleterious effects on healthy cells and resistance mechanisms to the BET inhibitors have been elucidated.

One of the major areas of interest regarding drug discovery is the great potential of combination therapies, especially in the case of resistance to existing standard therapy and or refractory states. Combination strategies, including pan-HDAC inhibitors in association with other agents and/or small molecules (chemotherapy, anti-GD2 antibody, retinoic acid, DNMTi, JQ1), are under evaluation in many pediatric and adult cancers. Specifically, the addition of JQ1 or EZH2 inhibitors to panobinostat (HDAC inhibitor) showed synergistic effects *in vitro* and *in vivo* (Shahbazi et al., 2016; Chen et al., 2018).

A detailed overview of the synergistic therapies with BET inhibitors and other epigenetic drugs or targeted agents can be found in Ramadoss and Mahadevan (2018). Aside from the above mentioned combination of HDAC and BET inhibitors, synergistic effects have also been demonstrated in combinatorial treatments using HDACs and DNMTs inhibitors, or DOT1L and DNMTs inhibitors in MLL-arranged leukemia cells (Klaus et al., 2014).

## Concluding Remarks

In the past few years, an improved survival rates in cancer patients has been witnessed, due to early diagnosis and the advent of new targeted therapies. However, there are still millions of patients who die every year. Tumor recurrence and relapse may be driven by a variety of molecular events that are modulated according to different treatment pressure. It is now clear that within the tumor bulk there is a subpopulation of cancer cells, named CSCs, which are mainly responsible for the anti-cancer drug refractoriness. Thus, the novel frontiers of cancer treatment are aimed at defeating CSCs by using newly discovered drug delivery methods. For instance, one appealing approach is represented by the use of nanotechnology as an efficient tool for detection and elimination of CSCs (Qin et al., 2017). Nanomaterials including gold particles, origami and tetrahedron DNA nanostructures, liposomes, graphene and nanodiamond have been loaded with chemotherapeutics compounds or agents effective against CSCs, such as Salinomycin and Hedgehog pathway inhibitors (Xu et al., 2012; Yao et al., 2014). The enzymatic functionalization of nanomaterials with ligands of cell surface markers of CSCs, such as CD44 and CD133, is crucial to confer specificity in CSCs binding and targeting (Yao et al., 2014; Ni et al., 2015; Al Faraj et al., 2016). The potential clinical application of these carriers rely also on their high solubility, fast internalization and photothermal features.

The urgent need of successful cancer cures is due to the mechanisms of CSC resistance, which are disparate and act at different levels, including activation of survival pathways, metabolic adaptation, epigenetic modifications and immune escape. All these aspects have been thoroughly investigated in this review with the aim of offering an overview and food for thought on the novel developed therapeutic strategies to improve cancer patients management.

## Author Contributions

AT, VV, MG, AC, and PB drafted the manuscript. MT and GS critically reviewed this manuscript.

## Conflict of Interest Statement

The authors declare that the research was conducted in the absence of any commercial or financial relationships that could be construed as a potential conflict of interest.

## Footnotes

1 http://www.cancer.gov/clinicaltrials NCT01587703
